# PCA3 noncoding RNA is involved in the control of prostate-cancer cell survival and modulates androgen receptor signaling

**DOI:** 10.1186/1471-2407-12-507

**Published:** 2012-11-06

**Authors:** Luciana Bueno Ferreira, Antonio Palumbo, Kivvi Duarte de Mello, Cinthya Sternberg, Mauricio S Caetano, Felipe Leite de Oliveira, Adriana Freitas Neves, Luiz Eurico Nasciutti, Luiz Ricardo Goulart, Etel Rodrigues Pereira Gimba

**Affiliations:** 1 Instituto Nacional do Câncer/Programa de Carcinogênese Molecular and Programa de Pós Graduação Stricto Sensu em Oncologia, Rio de Janeiro, Brazil; 2 Universidade Federal do Rio de Janeiro, Rio de Janeiro, Brazil; 3 Universidade Federal de Goiás, Campus de Catalão, Goiás, Brazil; 4 Instituto de Genética e Bioquímica, Laboratório de Nanobiotecnologia, Universidade Federal de Uberlândia, Uberlândia, Minas Gerais, Brazil; 5 Department of Medical Microbiology and Immunology, University of California-Davis, Davis, CA, USA; 6 Universidade Federal Fluminense, Rio de Janeiro, Rio de Janeiro, Brazil; 7 Departamento Interdisciplinar, Universidade Federal Fluminense-PURO, Rua Recife s/n, CEP: 28890-000, Rio das Ostras, Rio de Janeiro, Brazil

**Keywords:** PCA3, Prostate cancer, Small interfering RNA, Cell survival, Noncoding RNA

## Abstract

**Background:**

PCA3 is a non-coding RNA (ncRNA) that is highly expressed in prostate cancer (PCa) cells, but its functional role is unknown. To investigate its putative function in PCa biology, we used gene expression knockdown by small interference RNA, and also analyzed its involvement in androgen receptor (AR) signaling.

**Methods:**

LNCaP and PC3 cells were used as *in vitro* models for these functional assays, and three different siRNA sequences were specifically designed to target PCA3 exon 4. Transfected cells were analyzed by real-time qRT-PCR and cell growth, viability, and apoptosis assays. Associations between PCA3 and the androgen-receptor (AR) signaling pathway were investigated by treating LNCaP cells with 100 nM dihydrotestosterone (DHT) and with its antagonist (flutamide), and analyzing the expression of some AR-modulated genes (TMPRSS2, NDRG1, GREB1, PSA, AR, FGF8, CdK1, CdK2 and PMEPA1). PCA3 expression levels were investigated in different cell compartments by using differential centrifugation and qRT-PCR.

**Results:**

LNCaP siPCA3-transfected cells significantly inhibited cell growth and viability, and increased the proportion of cells in the sub G0/G1 phase of the cell cycle and the percentage of pyknotic nuclei, compared to those transfected with scramble siRNA (siSCr)-transfected cells. DHT-treated LNCaP cells induced a significant upregulation of PCA3 expression, which was reversed by flutamide. In siPCA3/LNCaP-transfected cells, the expression of AR target genes was downregulated compared to siSCr-transfected cells. The siPCA3 transfection also counteracted DHT stimulatory effects on the AR signaling cascade, significantly downregulating expression of the AR target gene. Analysis of PCA3 expression in different cell compartments provided evidence that the main functional roles of PCA3 occur in the nuclei and microsomal cell fractions.

**Conclusions:**

Our findings suggest that the ncRNA PCA3 is involved in the control of PCa cell survival, in part through modulating AR signaling, which may raise new possibilities of using PCA3 knockdown as an additional therapeutic strategy for PCa control.

## Background

The noncoding RNA PCA3, which was initially characterized as Differential Display Code 3 (DD3), is prostate-tissue-specific and highly overexpressed in more than 95% of primary prostate cancers [1]. PCA3 expression has shown promising applications for PCa diagnosis in urine samples after intense prostate massage [2,3], and also in blood and tissue samples [2,4,5]. Combining PCA3 with other new biomarkers further improves diagnostic and prognostic accuracy [6-9]. The specific activity of the PCA3 promoter in PCa cells may also be used as an additional strategy for targeted therapeutic approaches [10].

The gene encoding PCA3 is located on chromosome 9q21-22 in antisense orientation within intron 6 of the Prune homolog 2 gene (PRUNE2 or BMCC1) [11]. Further characterization of the PCA3 transcript sequence revealed alternative splicing and alternative polyadenylation, and due to a very short open reading frame, it was designated as a non-coding RNA (ncRNA) [1].

The PCA3 polyadenylation and its nuclear expression pattern support the hypothesis of a functional role in prostate biology [12]. However, other investigators have pointed out that PCA3 is also expressed in the cytoplasm of tumor cells and is not expressed in the stromal compartment [13]. The upregulation of PCA3 expression in PCa tissues seems to be an early event in prostate-tumor development, since its expression has been observed in almost all types of PCa tissue samples that have been analyzed, including well-differentiated, moderately differentiated, and poorly differentiated tumors [1]. Additionally, PCA3 expression seems to be restricted to cell lines that express androgen receptor (AR), such as LNCaP cells [14]. Although broadly characterized as a PCa specific biomarker, to our knowledge, data on the roles of PCA3 in PCa biology and tumor progression have not yet been provided.

Many ncRNAs are highly expressed and specifically regulated in tumors, which argues in favor of their functional significance. MicroRNAs are the best-known ncRNAs, although many other long ncRNAs exist. Different approaches have been used to investigate the putative biological roles of ncRNAs, including transcript overexpression, mutagenesis, DNA/RNA ChiP and gene knockdown techniques. Among these, *in vitro* and *in vivo* gene knockdown approaches, such as antisense oligonucleotides and RNA interference, are the main strategies used to investigate the roles of ncRNAs [15]. Herein, by using small interfering RNA to knock down PCA3 gene expression in PCa cells, we provided evidence that PCA3 is involved in PCa cell survival, which may be partially modulated by the androgen-receptor pathway.

## Methods

### Cell culture

LNCaP and PC3 prostate-cancer cell lines were obtained from ATCC (Rockville, MD, USA) and maintained in RPMI-1640 medium (Sigma) supplemented with 10% heat-inactivated fetal bovine serum (FBS) (Invitrogen/Life Technologies, Inc.). The RWPE-1 non-tumorigenic immortalized prostate cell line was a generous gift from Dr. Carlos Moreno (Emory University, USA) and was maintained in Keratinocyte-Serum-Free (KSF) (Invitrogen) supplemented with EGF (epidermal growth factor) and BPE (bovine pituitary extract). The PrEC, a non-tumoral primary prostate cell line (Cambrex BioScience, Walkersville, MD, USA) was maintained in PrEGM™ Prostate Epithelial Cell Growth Medium according to the supplier’s protocol. The DU145 cell line was obtained from ATCC and maintained in Dulbecco's Modified Eagle's Medium (DMEM) (Invitrogen) with 10% FBS. HeLa and NIH3T3 cell lines were cultured in DMEM containing 10% FBS. All these cell lines, except PrEC, were cultured in the presence of 100 U/mL penicillin and 100 μg/mL streptomycin. Cell cultures were maintained at 37°C in a 5% CO_2_ humidified incubator. Primary prostate stromal cells were isolated and characterized as follows. Transurethral resection fragments of prostate tissues obtained from three PCa surgeries were used to obtain the stromal cells. This procedure was approved by the Ethics Committee of Clementino Fraga Filho University Hospital, Federal University of Rio de Janeiro, and registered under protocol-CAAE 0029.0.197.000-05. Fragments of 1 to 3 mm^3^ were grown in 24-well plates containing DMEM (Sigma) culture medium supplemented with 10% FBS and standard antibiotics. The medium was changed every two days. After the cells attached to the bottom of the culture plate, they were trypsinized and then transferred to 25 mm^2^ culture dishes. After six passages, a homogeneous stromal cell population was established.

### PCA3 Expression knockdown by siRNA

Small interfering RNAs targeting the exon 4 of the PCA3 ncRNA (siPCA3) and a scramble siRNA sequence (siScR) were designed and synthesized by IDT Technologies. Sequences of these siRNAs were as follows:

siPCA3/1: 5'Phos/rGrCrArGrArArGrCrCrArGrArArUrUrUrGrArArUrUrCrCCT siPCA3/2: 5'Phos/rCrUrArGrCrArCrArCrArGrCrArUrGrArUrCrArUrUrArCGG

siPCA3/3: 5'Phos/rCrCrArCrArArUrArUrGrCrArUrArArArUrCrUrArArCrUCC

siScr: 5'Phos/rGrCrArCrGrCrUrCrCrUrArCrGrArArUrGrCrUrArGrUrArArA

All siRNAs were affinity-purified and annealed before use. On the day before transfection, LNCaP cells were plated in 2.0 mL of RPMI without antibiotics and supplemented with 0.5% FBS at a density of 2.5 × 10^5^ cells/6-well dishes. After 24 h, 500 μL of RPMI medium in each well was replaced by a combination of 60 nM siRNA solution, OPTi-MEM and Lipofectamine 2000 (Invitrogen), as described elsewhere [16]. The cells were maintained in culture for 36 h, and PCA3 knockdown expression was analyzed by real-time qRT-PCR, using 510R and 69F oligonucleotide sequences (Table 1).

**Table 1:** Oligonucleotide primers used for analysis of RT-PCR and qRT-PCR expression of androgen receptor-responsive genes and PCA3 transcript

***Gene***	***Primer 5’ – 3’***
PMEPA1-F	CATGATCCCCGAGCTGCT
PMEPA1-R	TGATCTGAACAAACTCCAGCTCC
18S-F*	AACCCGTTGAACCCCATT
18S-R*	CGCTACTACCGATTGGATGG
GAPDH-F*	TGACCCCTTCATTGACCTCA
GAPDH-R*	AGTCCTTCCACGATACCAAA
TMPRSS2-F	CTGGTGGCTGATAGGGGATA
TMPRSS2-R	GGACAAGGGGTTAGGGAGAG
NDRG1-F	CGAGACTTTACATGGCTCTG
NDRG1-R	GCATTGATGAACAGGTGCAG
GREB1-F	AAGGAGGGCTGGAAACAAAT
GREB1-R	CATTGTGGCCATTGTCATCT
PSA-F	TGCATCAGGAACAAAAGCGTGA
PSA-R	CCTGAGGCGTAGCAGGTGGTCCCCAG
AR-F	CCACTCGTCTCACGGGATAG
AR-R	GAAGACCTTGCAGCTTCCAC
FGF8-F	CAACTCTACAGCCGCACCAGC
FGF8-R	TGCTCTTGGCGATCAGCTTC
Cdk1-F	AAGTGAAGAGGAAGGGGTTCC
CdK1-R	CCAAAAGCTCTGGCAAGGCC
CdK2-F	ATGGGTGTAAGTACGAACAGG
CdK2-R	TTCTGCCATTCTCATCGG
PCA3 : 69-F	AGATTTGTGTGGCTGCAGC
PCA3 : 510-R	TCCTGCCCATCCTTTAAGG

*Also shown are the oligonucleotides used for analysis of constitutive gene expression.

**Figure 1:**
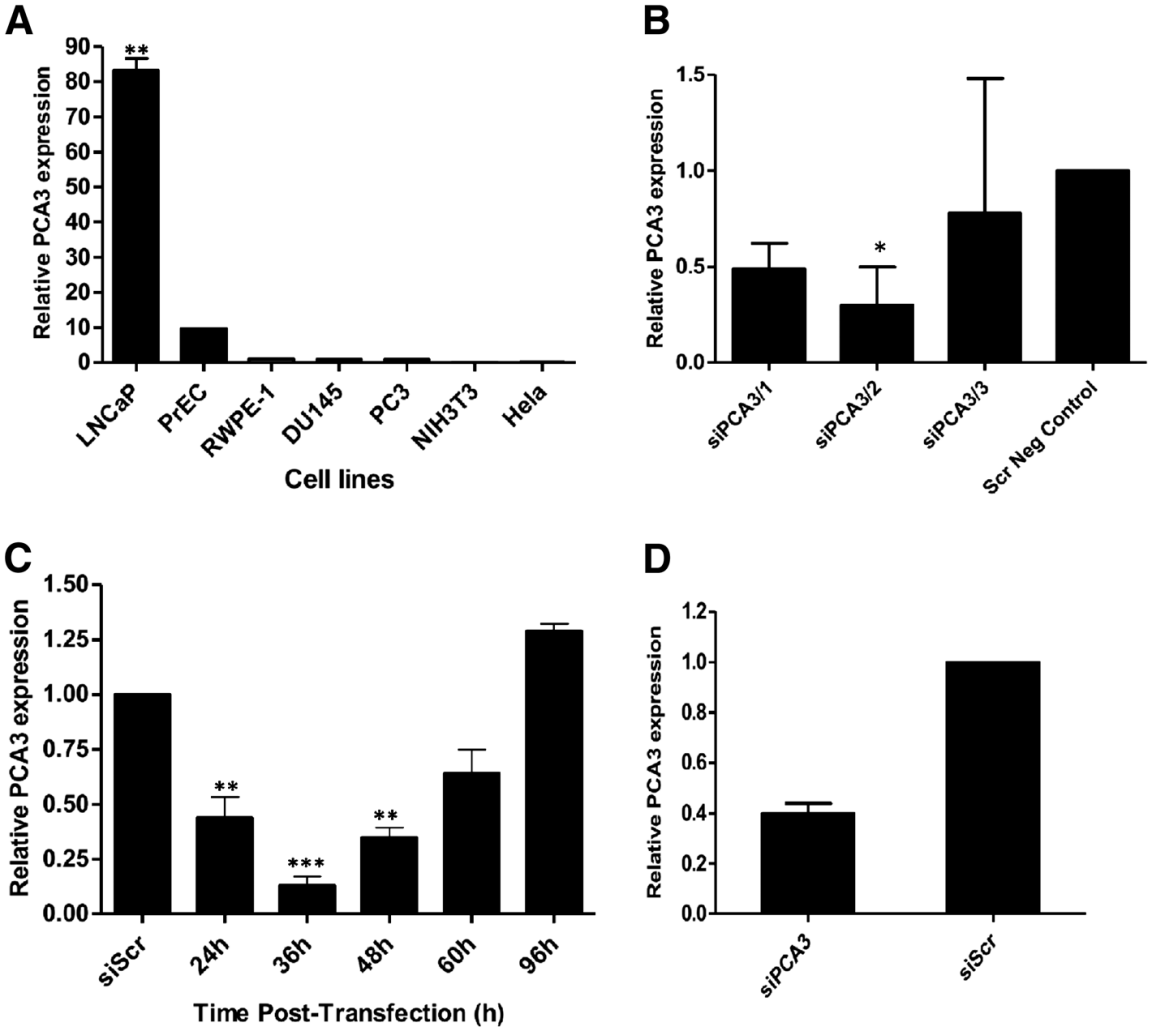
**Analysis of gene expression of PCA3 transcript in different cell lines and its targeted knockdown by siPCA3 in PCa cells.** (**A**) RNA expression of PCA3 was quantified by qRT-PCR in different prostate (LNCaP, PrEC, RWPE-1, DU145, and PC3) and non-prostate cell lines (NIH3T3 and HeLa). PCA3 expression was determined using the oligonucleotide primers described in the Methods section. PCA3 relative expression levels were determined in each cell line and compared to PCA3 expression in the DU145 cell line, used as a reference in this assay. (**B**) PCA3 expression was evaluated in LNCaP cells after knockdown using three different siPCA3 RNA sequences, termed siPCA3/1, siPCA3/2, and siPCA3/3. PCA3 expression was evaluated at 36 h post-transfection, and its relative expression level was determined compared to LNCaP cells transfected with the siSCr sequence, used as a negative control in this assay. (**C**) Following transfection of LNCaP cells with siPCA3/2, transcript levels were evaluated by qRT-PCR assays at the indicated time points. PCA3 relative expression is shown compared to siScr/LNCaP transfected cells. (**D**) PC3 cells were also transfected with siPCA3/2, and PCA3 expression was evaluated by qRT-PCR after 36 h post-transfection. 18S RNA was used as a constitutive gene. Data are shown as mean ± SD. All experiments were biological replicates, repeated a minimum of three times. **, p < 0.0017 and *** p < 0.0008.

Preliminary analysis of siPCA3/1 and siPCA3/3 demonstrated lower efficiencies for PCA3 knockdown in LNCaP cells after 36 h post-transfection (Figure 1B), compared to the siPCA3/2. Therefore, due to the higher siPCA3/2 efficiency in PCA3 silencing, all the remaining experiments were subsequently performed with the siPCA3/2, which is here termed siPCA3.

### Total RNA isolation and reverse transcription

Total RNA from all cultured cells was purified with the RNeasy Mini Kit (Qiagen) and treated with RNase-free DNase (Qiagen) during the RNA purification process. One microgram of RNA was reverse-transcribed using a “Superscript II First-Strand Synthesis System for RT-PCR” cDNA Synthesis kit (Invitrogen).

### Quantitative real-time PCR

Quantitative real-time PCR (qRT-pCR) was performed using a CFX96 Real-Time System (BIORAD) C1000 Thermal Cycler, the cDNA from all cultured cells, and Sybr Green (Applied Biosystems) as fluorophore, following the manufacturer’s instructions. Oligonucleotide primers used for qRT-PCR are listed in Table 1. The expression levels of ncRNA PCA3 and GAPDH, TMPRSS2, NDRG1, GREB1, PSA, AR, FGF8, CdK1, CdK2 and PMEPA1 mRNA levels were normalized based on the reference gene, to 18S rRNA, using the ΔΔCT. Conditions for PCR amplification were as follows: 50°C (2 min), 94°C (5 min) followed by 40 cycles at 94°C (30 s), 50°C (30 s) 72°C (45 s), and a final extension at 72°C (15 min). To evaluate the specificity of PCR products, a melting curve analysis was performed after each reaction.

### Cell growth and viability assays

Cell growth was analyzed by crystal violet staining after transfection of LNCaP, PC3, NIH3T3 and HeLa cells with siPCA3 and siScr, and the viability of LNCaP and PC3 cells was evaluated by trypan blue staining exclusion assay. The crystal violet assay was conducted by fixing cells in ethanol for 10 min, staining with 0.05% crystal violet for 10 min, and solubilizing with methanol. The supernatant was collected and its absorbance measured in an ELISA reader (BIO-RAD iMARKE) at 595 nm. LNCaP cells were pelleted by centrifugation and resuspended in 300 μL of phosphate-buffered saline (PBS, 1x solution) for viability analysis. Trypan blue (0.4% in PBS; 10 μL) was added to a 10 μL aliquot of cell suspension, and the number of viable unstained cells was counted using a haemocytometer.

### Cell cycle analysis

The cell cycle was analyzed by quantifying the amount of stained DNA with flow cytometry after transfection of LNCaP cells with siPCA3 and siScr. Approximately 2.5 × 10^5^ cells were incubated with 60 nM siPCA3 or siScr at the indicated time points. Cells were collected and resuspended in 200 μL of propidium iodide solution (PBS, 0.1% Triton X-100, 0.1% RNase, and 50 μg/mL propidium iodide; Sigma) and incubated for 5 min on ice. Cells were run on a BD FACSCalibur flow cytometer system (Becton Dickinson, Franklin Lakes, NJ, USA) with an absolute count of 20,000 cellular events. Data were obtained and analyzed with Cell-Quest 3.0.1 (Becton Dickinson, Franklin Lakes, NJ, USA) software.

### DAPI staining assay

LNCaP cells were plated in 2.0 mL of RPMI without antibiotics at a density of 2.5 × 10^5^ cells/6-well dishes containing a coverslip. After 24 h, 500 μL of RPMI medium of each well was replaced by a combination of 60 nM siRNA solution, OPTi-Mem, and Lipofectamine 2000 (Invitrogen). The medium was replaced after 8 h by 2.0 mL of RPMI containing 10% FBS and antibiotics (100 U/mL penicillin and 100 μg/mL streptomycin). The cells were maintained in culture for 36 h. Apoptotic cells were identified by the appearance of pyknotic nuclei. Morphological changes were determined by DAPI (4',6-diamidino-2-phenyl-indole, Molecular Probes, Eugene, OR, USA) staining. On the coverslips, 36 h after siPCA3 or siScr-LNCaP transfection, cells were fixed with 4% methanol-free formaldehyde solution for 30 min. Then, mounting medium with DAPI was dispersed over the entire slide section. Mounted slides were stored at 4°C without light. Each slide was observed under a 4.0 fluorescence microscope at 100x magnification.

### Androgen stimulation procedures

LNCaP cells were plated in 2.0 mL of RPMI without antibiotics at a density of 1.5 × 10^5^ cells/6-well dishes, and maintained in medium containing charcoal/dextran stripped FBS (CCS) (Invitrogen) for 3 days before treatment with dihydrotestosterone (DHT), flutamide, or the control vehicle (ethanol). DHT (Sigma, St. Louis, MO, USA) was dissolved in absolute ethanol at a concentration of 1 M and reconstituted in culture medium at a concentration of 100 nM in 0.5% ethanol. Culture medium containing 100 nM flutamide and 0.5% ethanol (Sigma, St. Louis, MO, USA) was prepared using the same procedure. The control medium contained only 0.5% ethanol. LNCaP cells were plated into 6-well culture plates and grown for 12 or 48 h before treatments with DHT or DHT plus 100 nM flutamide. Flutamide was added to cells 15 min before the 100 nM DHT treatment. LNCaP cells were grown until the indicated time points, and then cells were harvested for analysis of PCA3 expression. For the analysis of expression of androgen-regulated genes (PCA3, AR, PSA, TMPRSS2, NDRG1, GREB1, FGF8, CdK1, CdK2 and PMEPA1), LNCaP cells were seeded into 6-well culture plates and grown for 36 h before the 100 nM DHT treatment. For the simultaneous stimulation with DHT and PCA3 silencing by siPCA3, LNCaP cells were maintained in CCS for 3 days before siPCA3 transfections, and DHT (100 nM) was added to the LNCaP cells after 6 h. siPCA3 transfections were prepared according to the conditions previously described for PCA3 knockdown by siPCA3. Finally, at 36 h after transfection, the LNCaP cells were harvested for analysis of gene expression.

### Cell fractionation

siRNAs transfected LNCaP cells (1 × 10^7^) were lysed with liquid nitrogen and homogenized in a 0.25 M sucrose solution buffered to pH 7.4 with 5 mM Tris–HCl. The homogenate was centrifuged as described elsewhere [17] to obtain four pellets (P1, P2, P3 and P4), which sedimented respectively at 300 × g (5 min), 1000 × g (10 min), 8000 × g (10 min) and 100,000 × g (60 min). Fraction P1 contained mainly nuclei and some vesicles. Fraction P2 also contained nuclei, mitochondria, dense bodies, and vesicles. Fraction P3 contained mainly mitochondria, with a few vesicles and dense bodies. Finally, fraction P4 contained free vesicles and microsomes. The pellets were resuspended individually in buffered sucrose solution and stored at - 15°C until use.

### Preparation of cell lysates and immunoblot

Extracts of cells transfected with siPCA3 and siScr were prepared with Cell Lysis Buffer (Cell Signaling Technology), sonicated, and cleared by centrifugation at 15,000 × g. Total protein concentration was measured using the BCA assay kit (BioRad), according to the manufacturer’s instructions. LNCaP cells transfected with siPCA3 and siScr control were cultured as previously described. Immunoblot was performed using 50 μg of protein extracts. PI3-Kinase activation was analyzed by the levels of Akt Ser^473^ phosphorylation, and Erk1/2 activation was analyzed by the levels of Thr202/Tyr204 phosphorylation. Membranes were incubated with anti-total Akt and Erk1/2, anti-phospho-Akt antibodies, and anti-phospho-Erk1/2 (Cell Signaling Technology). Horseradish peroxidase (HRP)-conjugated anti-rabbit IgG (Pierce, Rockford, IL, USA) was diluted 1:1000 in PBST containing 5% bovine serum albumin. Chemiluminescence detection (Amersham Biosciences, USA) was performed according to the manufacturer’s instructions.

### Statistical analysis

Results are presented as the mean +/- standard deviation of at least three independent experiments. Differences among groups were evaluated by Student’s t-test, using GraphPad Prism software (San Diego, CA, USA). A value of *P* < 0.05 was considered to be statistically significant.

## Results

### Establishing a cell-line model to study the role of PCA3 in PCa

In order to perform *in vitro* functional assays to determine the putative role of PCA3 in PCa, we first analyzed the PCA3 transcript expression in different prostate (LNCaP, PrEC, RWPE-1, DU145 and PC3) and non-prostate (NIH3T3 and HeLa) cell lines. We analyzed the PCA3 transcript levels in order to determine which was the most appropriate model for further analysis. Consistent with previous reports [1,14], we found that PCA3 is highly expressed in the androgen-dependent prostate cell line LNCaP (Figure 1A), compared to the prostate cell lines PrEC, RWPE-1, PC3 and DU145. PCA3 expression was not detected in the non-prostate cell lines, NIH3T3 and HeLa. Our data have indicated LNCaP cells as the most suitable model to investigate the PCA3 function in PCa biology by using small interfering RNA to silence this ncRNA, which was upregulated in these cells when compared to other tested cells lines. We tested on LNCaP cells, three different specific siRNAs for PCA3, termed siPCA3/1, siPCA3/2, and siPCA3/3, and evaluated their efficiencies for PCA3 knockdown. Because of the significant effect on PCA3 silencing by the siPCA3/2 (Figure 1B), we thereafter used this siRNA (siPCA3) molecule to perform all the functional assays. The PCA3 RNA levels decreased progressively from 24 h to 36 h post-transfection in the siPCA3/2-LNCaP transfected cells, with a marked loss at 24 h (Figure 1C), compared to the siScr-transfected LNCaP, used as a negative control. The maximum loss of PCA3 expression was observed at 36 h post-siPCA3 transfection. PCA3 expression was restored after 48 h post-transfection, indicating siPCA3 withdrawal. We also evaluated the PC3 cell transfection with siPCA3, in order to determine the effect of the PCA3 knockdown in a hormone-independent PCa cell line, which shows lower levels of PCA3 expression than the LNCaP cells. PCA3 expression of the PC3 cell line was also significantly inhibited at 36 h post-siPCA3 transfection (Figure 1D). These data further reinforce the effect of siPCA3 in silencing PCA3 expression, in both hormone-dependent and hormone-independent cell lines.

### PCA3 silencing decreases cell growth and survival and induces apoptotic cell death in prostate-cancer cells

**Figure 2:**
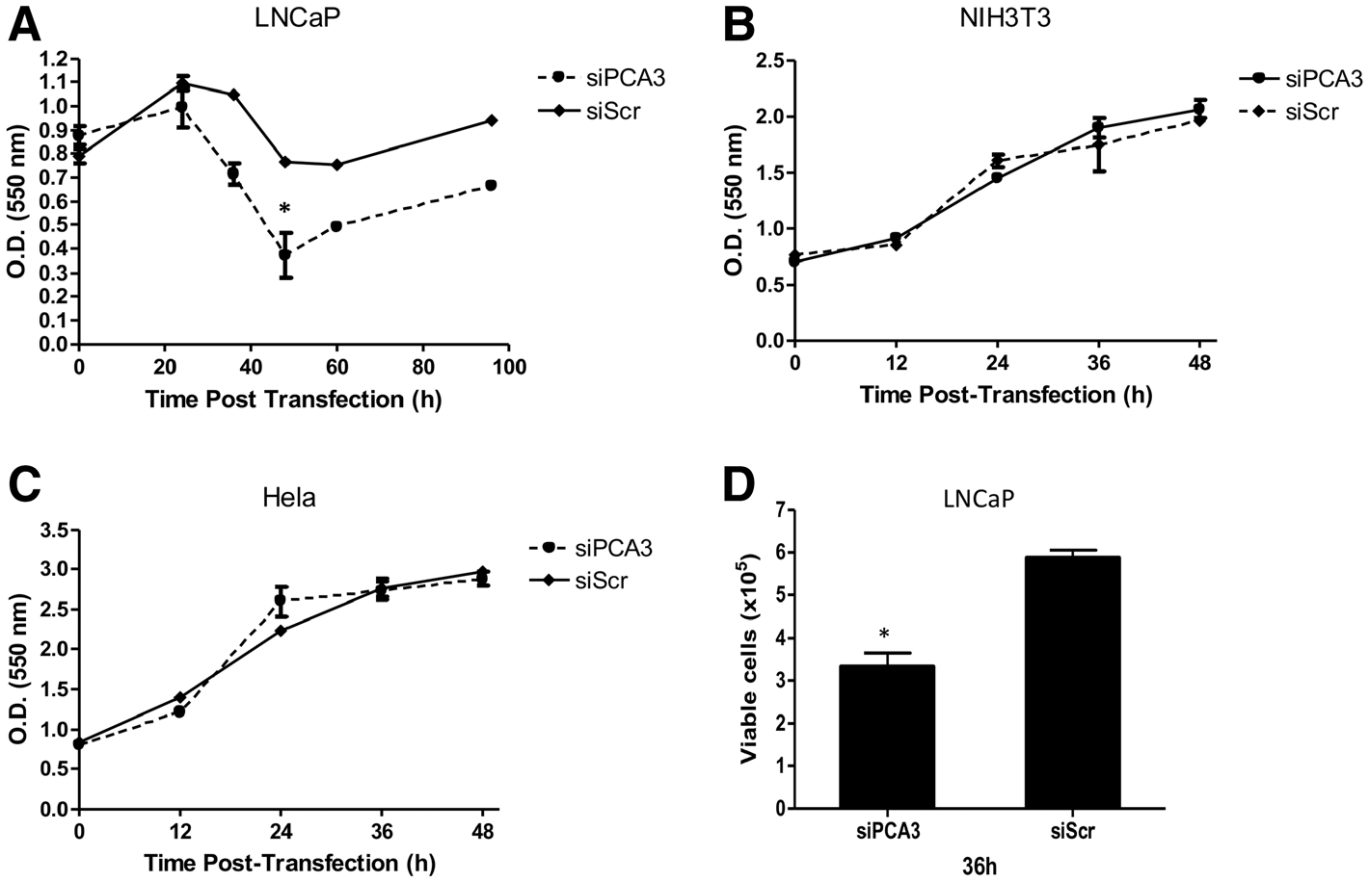
**PCA3 knockdown inhibits LNCaP cell growth and survival.** (**A**) LNCaP cells were transfected with 60 nM of siPCA3 and non-targeting siRNA (siScr), and then followed for cell proliferation for 96 h. (**B**) NIH3T3 and (**C**) HeLa cells were transfected with 60 nM of siPCA3 or non-targeting siRNA (siScr) and then followed for cell growth for 48 h. Cell growth was evaluated using the crystal-violet assay described in the Methods section. Data represent the mean ± SD of three independent experiments (* p < 0.05). (**D**) The effect of PCA3 knockdown on LNCaP cell viability was measured using Trypan Blue exclusion analysis. Data are shown as the mean ± SD of three experiments.

A number of hallmark events are associated with tumor progression, of which apoptosis evasion and increased cell proliferation are among the most critical ones. Our first approach to investigate the putative roles of PCA3 in PCa biology was to determine LNCaP cell growth rates by using crystal-violet assays over a 100-h time course after LNCaP cell transfection with siPCA3 (Figure 2A). After 24 h, siPCA3-transfected LNCaP cells progressively decreased cell growth, compared to siScr-transfected LNCaP cells. This decrease in cell growth was maintained until 50 h post-siPCA3 treatment, when cell growth was restored, possibly as a consequence of siPCA3 withdrawal, coinciding with the PCA3 silencing expression profile (Figure 1C). Interestingly, siScr-transfected LNCaP cells showed no significant increase in cell growth during the time-course evaluation, suggesting that the decreased cell growth after PCA3 knockdown in siPCA3-transfected cells may be due to an effect on cell viability rather than on cell proliferation.

We then asked whether this observed decrease in cell growth as a result of PCA3 silencing was specific for cells expressing PCA3 transcript, and also attempted to exclude the possibility of siPCA3 off-target effects. NIH3T3 and HeLa cells, which did not express PCA3 (as shown in Figure 1A), exhibited no change in cell growth rates after siPCA3 transfection on a 60-h time course evaluation (Figure 2B and 2C). These data further indicated that PCA3 knockdown is specifically inhibiting the growth rates of PCA3-expressing cells, and that the siPCA3 molecule is specifically targeting PCA3.

**Figure 3:**
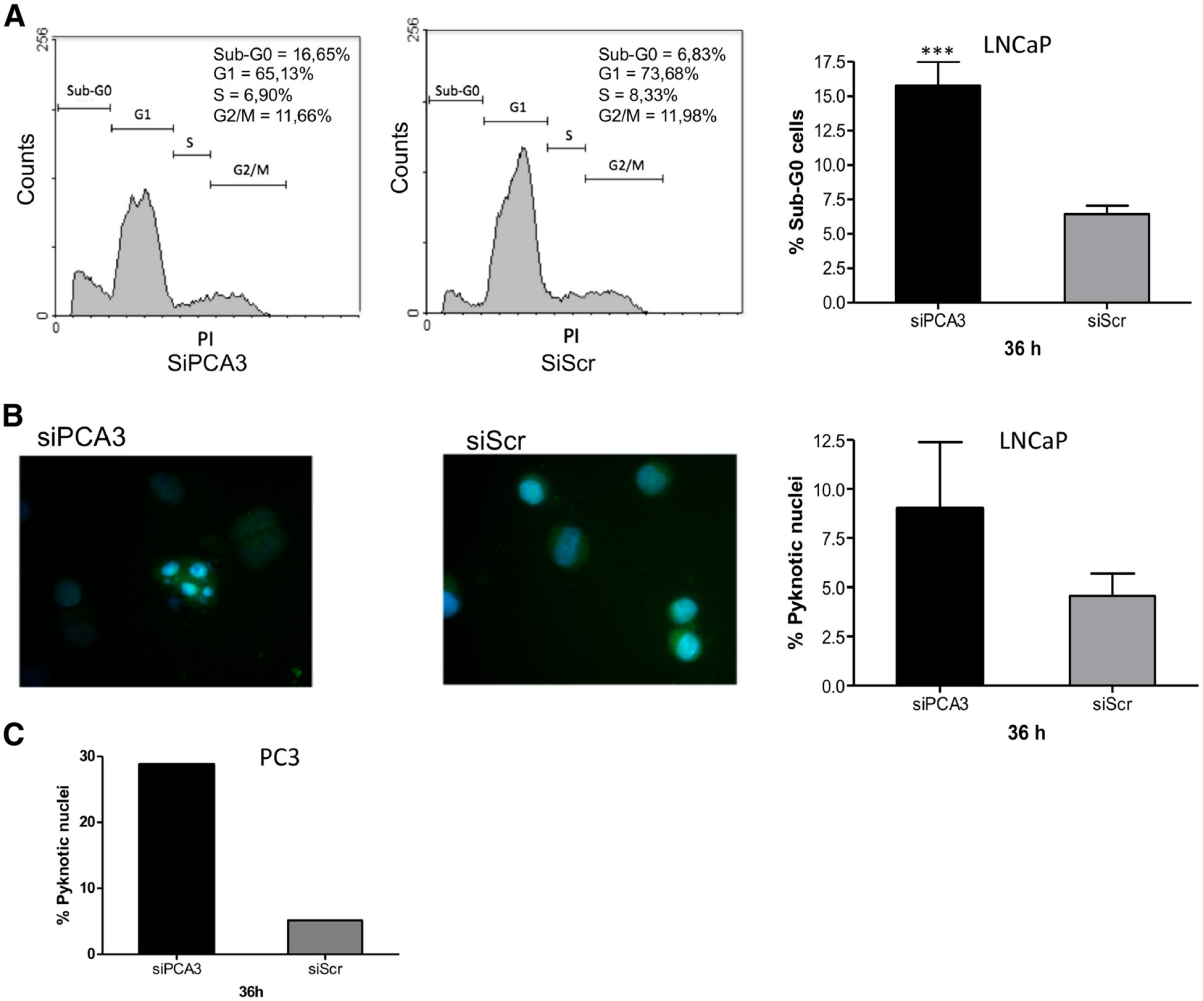
**PCA3 knockdown induces cell cycle arrest and apoptosis in LNCaP cells.** (**A**) Representative histograms of LNCaP cells 36 h after transfection with siPCA3 or siScr (left panels). Flow cytometry was used to quantify the percentage of cells undergoing apoptosis (cells in sub G0-G1), which are shown in the bar graph on the right panel. The data represent the means ± S.D. from three independent experiments (right panel). The asterisks indicate significant differences between siPCA3 and siScr control-treated groups (*** p < 0.001). (**B**) Nuclear morphological changes characteristic of apoptotic cells in LNCaP cell cultures transfected with siPCA3 or siScr (left panels) were analyzed. A representative experiment is shown. Following fixation and staining with DAPI, LNCaP cells transfected with siPCA3 or siScr were examined and photographed using a fluorescence microscope as described in the Methods section. The number of pyknotic nuclei in each experimental sample was counted, and is represented on the bar graph in the right panel as the percentage of cells with pyknotic nuclei in relation to the total number of cells counted. (**C**) Nuclear morphological changes characteristic of apoptotic cells in PC3 cell cultures transfected with siPCA3 or siScr (left panels) were analyzed, and the results represented as described for LNCaP cells in (**B**). The data represent the means ± S.D. from three independent experiments (right panel).

We also observed that LNCaP cell viability was significantly reduced in siPCA3-transfected LNCaP cells, compared to siScr-transfected cells (Figure 2D), as measured by trypan-blue exclusion assays at 36 h post-siRNA transfection. Moreover, LNCaP-transfected cells with either siPCA3 or siScr were monitored by flow cytometry stained with propidium iodide, which allowed the examination of intra-culture populations in different cell cycle phases. LNCaP cells transfected with siPCA3 showed a significant increase in the proportion of cells in the G_0_ phase at 36 h post-transfection when compared to siScr-LNCaP transfected cells (Figure 3A), which is an indication of cells undergoing apoptosis. Further evidence of apoptosis in siPCA3-transfected cells was obtained by fluorescence microscopy after DAPI DNA-staining (Figure 3B), which showed a higher percentage of pyknotic nuclei in siPCA3-LNCaP (9.04%) than in siScr-LNCaP cells (4.56%). Therefore, the PCA3 knockdown in LNCaP cells may be associated with a higher proportion of cells undergoing apoptosis, and suggests that PCA3 may also be able to modulate PCa cell survival. We also tested the effect of PCA3 silencing on PC3 cell viability. These cells also showed a significant increase in the percentage of cells containing pyknotic nuclei, compared to siScr–PC3 transfected cells (Figure 3C). These data further reinforce the notion that PCA3 modulates PCa cell survival, specifically of PCA3-expressing cells.

### PCA3 expression is upregulated by androgen-receptor signaling, and modulates the expression of AR target genes

Pro-survival signaling mediated by the androgen receptor (AR) is implicated as a key contributor to prostate carcinogenesis, which classically controls PCa cell proliferation, survival, and differentiation [18,19]. Considering that PCA3 expression is upregulated in androgen-dependent cell lines, such as LNCaP cells, we investigated the putative involvement of PCA3 in regulating the AR pro-survival signaling pathway.

**Figure 4:**
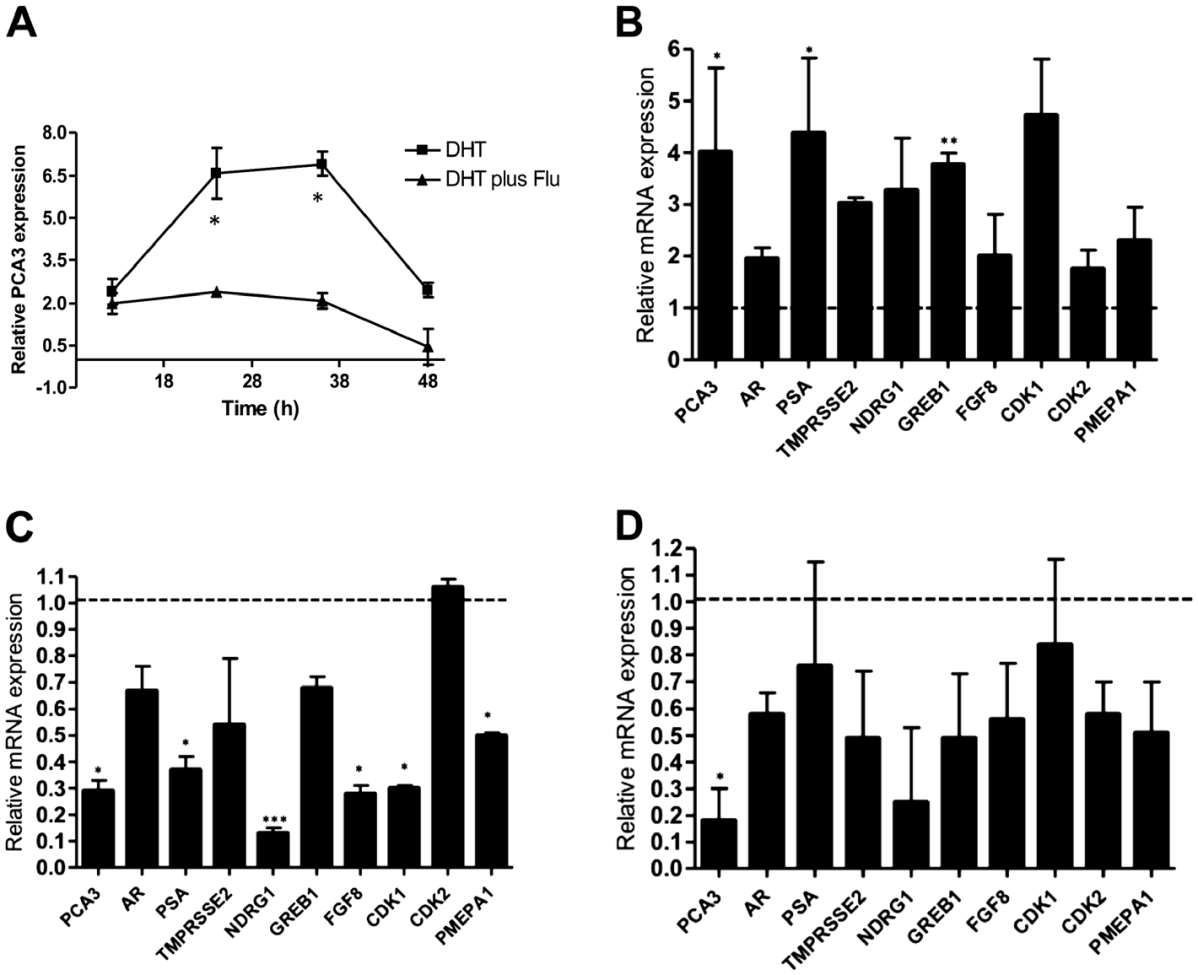
**PCA3 expression is upregulated by androgen and modulates the transcription of androgen-regulated genes.** (**A**) PCA3 expression was evaluated in LNCaP cells by qRT-PCR after treatment with 100 nM of DHT or 100 nM DHT plus 100 nM flutamide during a 48-h time course, as described in the Methods section. PCA3 relative expression was determined compared to LNCaP cells treated with ethanol, which was the control vehicle. Error bars +/- SD. (**B**) Relative RNA quantification of PCA3, AR and androgen-regulated genes (TMPRSS2, NDRG1, GREB1, PSA, AR, FGF8, CdK1, CdK2, and PMEPA1) in LNCaP cells treated with 100 nM of dihydrotestosterone (DHT) for 36 h, compared to cells treated with the control vehicle (ethanol), as described in the Methods section. Bar graphs show the average transcript levels of each gene tested, by qRT–PCR analysis of three independent RNA samples prepared following the treatment of LNCaP cells with DHT or ethanol only. Error bars +/- SD. (**C**) Relative RNA levels of PCA3, AR, and androgen-regulated genes 36 h after LNCaP cells were transfected with siPCA3, compared to LNCaP/siSCr transfected cells. Error bars +/- SD. (**D**) Relative RNA levels of PCA3, AR, and androgen-regulated genes 36 h after LNCaP cells were transfected with siPCA3 simultaneously with treatment with 100 nM DHT, compared to LNCaP cells transfected with siScr simultaneously with treatment with 100 nM DHT, as described in the Methods section. 18S RNA was used as a constitutive gene in all these assays. Data are represented as mean ± SD. All experiments were biological replicates repeated a minimum of three times. *, p < 0.01, in comparison to scrambled-siRNA (siScr) treated cells.

To elucidate how PCA3 could be related to AR pro-survival signaling, we first demonstrated the PCA3 expression response of LNCaP cells submitted to androgen stimulation, and then the involvement of AR in this signaling. Previous reports demonstrated that PCA3 expression is responsive to DHT stimulation [11,12,20]. However, it has not been clearly demonstrated whether the activated AR mediates this androgen-responsive PCA3 expression. We first evaluated PCA3 expression levels in LNCaP cells treated with dihydrotestosterone (DHT), the androgen active metabolite, for 48 h. Corroborating other reports, we also found that DHT-stimulated LNCaP cells significantly activated PCA3 expression (Figure 4A). To demonstrate that the upregulated PCA3 expression was directly controlled by the signal mediated by the activated AR, we treated these cells with DHT together with an AR antagonist flutamide. PCA3 upregulation evoked by DHT was reversed by flutamide, since it competes with DHT for AR binding. These data demonstrated that PCA3 expression is androgen-regulated via the activated AR-mediated signal. The increased AR transcriptional activity was confirmed by the upregulation of all AR-responsive genes after DHT treatment, including the increased expression of AR and PCA3 (Figure 4B). Although the upregulation was not statistically significant for all genes tested, they showed at least a 1.5-fold increase in their expression levels when compared to non-DHT-treated LNCaP cells. Five of the eight AR-responsive genes tested showed at least a 3-fold increase in their expression levels after DHT treatment. AR and PCA3 transcript expressions were also upregulated in these experimental conditions. Considering that the androgen-responsive LNCaP cell line expresses higher PCA3 levels than the other cell lines tested, and that downregulation of this ncRNA significantly decreases LNCaP survival, we then speculated whether PCA3 silencing could modulate the expression of AR target genes, and whether this event could be related to the observed decrease in LNCaP survival. LNCaP cells transfected with siPCA3 caused a significant downregulation of seven of the eight AR-regulated genes tested, compared to the cells transfected with siScr (Figure 4C). Five of these downregulated genes showed a statistically significant decrease in their expression levels (p< 0.05): PSA, NDRG1, FGF8, CDK1, and PMEPA1. Then, we analyzed the effect of PCA3 silencing together with DHT stimulation. The upregulation of the AR target genes triggered by the DHT treatment was reversed by the concomitant PCA3 knockdown (Figure 4D). Six of eight AR target genes tested showed at least a 40% decrease in their expression levels, including TMPRSSE2, NDRG1, GREB1, FGF8, CDK2, and PMEPA1, although this decrease was not statistically significant (p>0.05). Both PCA3 and AR transcription levels were also downregulated in this condition. These results suggest that PCA3 is somehow modulating the expression of AR signaling, which should be further investigated. In summary, our data suggest that the ncRNA PCA3 is responsive to AR signaling and may act as a transcriptional modulator of the AR target genes.

**Figure 5:**
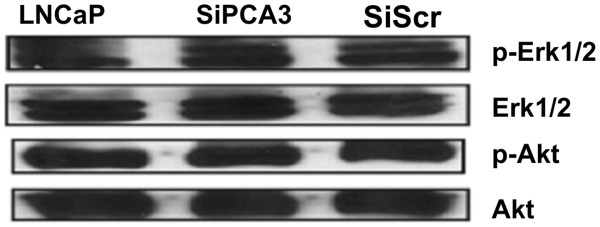
**Downregulation of PCA3 expression does not significantly reduce Akt and Erk1/2 phosphorylation in LNCaP cells.** The Akt and Erk1/2 phosphorylation profile before and after transfection of LNCaP cells with either siPCA3 or siScr was investigated by immunoblot analysis using 50 μg of total protein extracts. Akt and Erk1/2 non-phosphorylated proteins were used as controls for normalization of protein loading.

We also evaluated whether key mediators of signaling pathways that cross-talk with the AR pathway were also modified in siPCA3-LNCaP transfected cells. It is well known that PI3K-Akt and MAPK signals mediate key pro-survival roles in PCa cells, and that their phosphorylated forms are able to modulate AR activation and the androgen-independent transcriptional activation of AR target genes. Activation of these signals promotes the phosphorylation of AR and its co-regulators, increasing AR transcriptional activity and the expression of AR target genes involved in PCa pro-survival roles [21,22]. The phosphorylation status of Akt and ERK was tested in the siPCA3-LNCaP cells, as representative genes of the growth-factor signaling pathways that cross-talk with AR signaling (Figure 5). We found that siPCA3-transfected LNCaP cells did not modify Akt and ERK phosphorylation levels compared to siScr-transfected LNCaP cells, suggesting that PCA3 may modulate LNCaP survival mainly through downstream signals of the activated AR signaling axis.

### PCA3 is mostly expressed in nuclear and microsomal cell compartments

**Figure 6:**
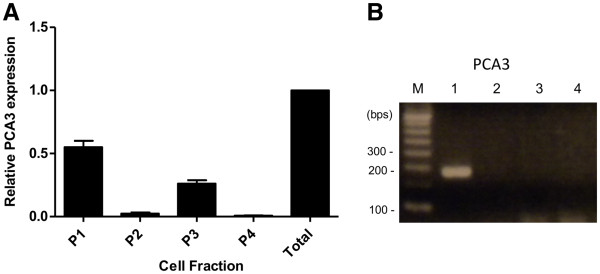
**PCA3 is mostly expressed in LNCaP nucleus and microsomal cell fractions.** (**A**) Total RNA samples obtained from different LNCaP cell compartments. Fraction P1 contained mainly nuclei and some vesicles. Fraction P2 contained nuclei, mitochondria, dense bodies, and vesicles. Fraction P3 contained mainly mitochondria, with a few vesicles and dense bodies. Finally, fraction P4 contained free vesicles and microsomes. Bar graphs represent PCA3 ncRNA relative expression levels in each subcellular compartment, compared to total RNA from LNCAP cell whole extracts, containing a mixture of RNA samples from all cell compartments. 18S rRNA was used as a constitutive gene. (**B**) Analysis of PCA3 expression by RT-PCR in total RNA samples from primary prostate stromal cell cultures. M: molecular weight marker, 100 bp; Lane 1: Total RNA samples from a single PCa tumor expressing PCA3; Lanes 2 – 4: Three different primary prostate stromal-cell cultures.

As an additional approach to investigate the putative roles of PCA3 in PCa cells, we determined the cell compartment localization of PCA3 in LNCaP cells by differential centrifugation and qRT-PCR. RNA was extracted from different cell compartments obtained through differential centrifugation, and PCA3 expression was analyzed. Our data showed that PCA3 expression was mainly restricted to the nuclear and microsomal compartments (P1 and P3 fractions, respectively) of LNCaP cells (Figure 6A). We also tested PCA3 expression in primary prostate stromal cell cultures, which showed no PCA3 expression (Figure 6B). Our results suggest that the nuclear and microsomal cell compartments of prostate-tumor epithelial cells are the major sites of PCA3 expression, where PCA3 may play its main roles in controlling PCa cell pro-survival features.

## Discussion

The PCA3 ncRNA is one of the most prostate-specific genes described to date, is highly overexpressed in PCa tumors, and has been extensively characterized as a tumor biomarker [1,2,4,7,23]. However, no function has been attributed to this transcript in PCa cells [1,2,4,7,23]. Our primary aim was to elucidate the putative roles of this ncRNA in PCa cell biology. Previous data have partially supported the concept that PCA3 is a functional transcript, as argued for other ncRNAs [1,12]. The limited expression of PCA3 in prostate tissues suggests that it may function specifically in this organ. This also suggests that its expression may be tightly regulated, as would be expected for a functional transcript. The results obtained here support the notion that PCA3 is involved in PCa survival pathways by controlling cell growth and viability, at least in part through controlling the AR pro-survival signaling. In addition, our results accord with the hypothesis that PCA3 is involved in transcriptional modulation of AR target genes, although it may act through a still-unknown mechanism.

Other ncRNAs have also been described as being involved in cancer cell survival, including PlncRNA-1, GAS5, HOTAIR, and several miRNA genes [24-27], which act by controlling apoptosis, the cell cycle, cell proliferation, or viability, through their interactions with intracellular signaling networks. Other ncRNAs involved in PCa cell survival and proliferation have also been described, including PCAT-1 [28], PRNCR1 [29], PCGEM1 [30], and PlncRNA-1 [24]. Similarly to PCA3, the AR signaling pathway also modulates the expression of PRNCR1, PlncRNA-1, and PCGEM1. Additionally, several androgen-responsive intronic ncRNAs have been described, indicating that intronic ncRNAs, such as PCA3 [31], may have control mechanisms that are common to protein-coding transcripts, such as those involving hormonal control of gene-promoter activation [32]. The facts that PCA3 is expressed in higher levels in the androgen-responsive cell line (LNCaP) and that some ncRNA expressed in PCa cells are involved in the AR signaling [24,29,30] led us to hypothesize that PCA3 ncRNA expression may also be modulated by this pathway, and that this transcript may also be involved in the control of genes related to this cell signaling.

Uncontrolled cell growth is a result of a progression of changes at the cellular and genetic levels, which ultimately reprogram a cell to undergo uncontrolled division, and is one of the first steps in carcinogenesis. In order to investigate the putative involvement of PCA3 in controlling this early step of tumorigenesis, we investigated the behavior of LNCaP cell growth after PCA3 knockdown by RNA interference. PCA3-silenced LNCaP cells showed a significant attenuation of cell growth, with a corresponding increase in the number of cells undergoing apoptosis. Transfected cells with siScr also showed a slight decrease in cell growth after 20 h post-transfection, which could be due to a cytotoxicity effect promoted by the lipofectamin transfection reagent and acting specifically on LNCaP cells, which has not been observed in other cell lines. Notably, siPCA3-LNCaP transfected cells show a greater decrease in cell growth rates than do siScr-transfected cells, indicating that PCA3 ncRNA is involved in PCa survival and that siPCA3 specifically targeted PCA3 transcripts, since growth rates were not modified in cells that do not express PCA3. Considering that PCA3 is specifically expressed in prostate tissues [1], it is possible that PCA3 silencing would be an interesting therapeutic approach, especially to inhibit PCa growth and progression, as has been proposed for other genes involved in PCa survival [33,34]. The evidence presented in this study, for a potential modulating role of PCA3 in the AR signaling, highlights the importance of this transcript as a potential target for treatment. This may also be an interesting approach during PCa progression, especially when androgen resistance is developed [35]. In addition, PCA3 expression at different PCa stages [1] further reinforces the notion that this ncRNA plays an essential role during PCa tumorigenesis and progression.

In order to further emphasize the role of PCA3 in modulating PCa cell survival, we also investigated the effect of PCA3 knockdown in an androgen-independent PCa cell line, which simulates a more aggressive PCa disease. However, conflicting results have been reported regarding the androgen independence of these cells and AR protein expression in the PC3 and DU145 cell lines, as well in castration-resistant prostate cancer tumors [36,37]. Although the majority of human prostate-cancer cell lines are reported to be AR-negative [38,39], several studies have indicated that the DU-145 and PC-3 prostate-cancer cell lines express detectable levels of the AR mRNA [40-44]. For this reason, the exact role of AR in PC3 cells is still controversial [36]. Although PC3 cells showed lower PCA3 expression than did the LNCaP cells, we asked whether PCA3 also regulates cell survival in these cells. Considering the heterogeneity of PCa tumors regarding gene expression profiling and tumor progression behavior, we then wanted to evaluate survival features after PCA3 knockdown even in cell lines that express low PCA3 levels, and that in principle are androgen-independent. Since PC3 is a well-established cell-line model representing a more aggressive stage of prostate cancer tumors, our data provide additional evidence for a role of PCA3 in modulating PCa cell survival. We also wanted to investigate how these androgen-independent cells would respond to PCA3 knockdown. Our results demonstrated that PCA3 knockdown, as demonstrated by the significant decrease in PCA3 levels after transfecting PC3 cells with the siPCA3 molecule, increased the proportion of cells with pyknotic nuclei (compared to PC3 transfected with siScr control), which was indicative of a larger number of cells undergoing apoptosis, further evidencing the role of PCA3 in modulating PCa cell survival, even in an androgen-independent cell-line model that expresses lower PCA3 levels compared to LNCaP cells. However, how PCA3 can modulate PCa cell survival in these cells and whether it is mediated by different mechanisms from those observed in siPCA3-LNCaP transfected cells, should be further investigated.

Due to the increased PCA3 expression in androgen-responsive cells compared with androgen-insensitive cells [36], and because AR signaling is an important pathway controlling PCa survival, we tested whether PCA3 expression was modulated by the androgen-active metabolite DHT and whether this expression pattern involved the activated AR. Upregulation of PCA3 expression in response to LNCaP stimulation with DHT was significantly counteracted by the AR antagonist flutamide, indicating that PCA3 expression was induced by the activated AR. AR activation was further confirmed by the observation that LNCaP cells stimulated with DHT also showed AR transcriptional activity. Consistently, all of the AR target genes tested that contain canonical AR response elements (AREs) in their promoter sequences, were upregulated upon DHT treatment. Although eight of the genes showed at least a 1.5-fold increase after AR activation, only two of them showed a significant increase in their expression levels. Interestingly, PCA3 upregulation upon DHT treatment has been observed previously [11,20], but no study has demonstrated the involvement of activated AR in PCA3 expression by using AR antagonists. Although our data also suggest that PCA3 is an androgen-responsive gene, the precise molecular mechanism by which PCA3 expression responds to this activation is still unknown. One hypothesis is that activated AR can directly activate the PCA3 promoter, as has been demonstrated for the miR-101 and miR-21 regulatory regions [45,46], which are also modulated by the activated AR. However, no consensus AREs have been identified in the 500-bp PCA3 promoter region [47]. We further screened for consensus ARE elements in the entire PCA3 genomic region at the 5 Kb region upstream from the PCA3 transcription start site, and have so far identified no canonical element (data not shown). Nevertheless, we cannot exclude the possibility that other, non-canonical ARE elements could also promote AR binding and directly activate PCA3 expression, as has been previously described for other genes modulated by the AR activation [48,49]. PCA3-upregulated expression in response to DHT treatment could also be a result of activated AR binding to the regulatory regions of other AR-responsive genes, which in turn could induce PCA3 expression. Further experiments should investigate direct AR binding to different PCA3 genomic regions, in order to answer these open questions.

Our data support a pro-survival role for PCA3, since its downregulation, in addition to inhibiting PCa survival, decreased the expression of AR target genes, most of them typically involved in androgen-dependent cell growth. The close association between the involvement of PCA3 in PCa cell survival and the modulation of the expression of AR should be further investigated, in an attempt to elucidate how PCA3 expression levels can regulate AR signaling and target genes. Furthermore, PCA3 knockdown inhibited the expression of all AR target genes tested, even under DHT treatment, indicating that the final effect of PCA3 downregulation may be stronger than the effect of DHT stimulation in modulating the expression of AR target genes. On the other hand, Akt and ERK phosphorylation levels remained unchanged in siPCA3-transfected cells, indicating that alternative pathways able to activate AR irrespective of ligand activation [50] were not altered after PCA3 knockdown. Taken together, these data suggest that the role of PCA3 in modulating the expression of these AR target genes may function downstream from AR activation.

As an approach to investigate the signal by which PCA3 controls PCa cell survival, we analyzed the transcript expression of PSA, AR, TMPRSS2, NDRG1, GREB1, FGF8, CDK1, CDK2, and PMEPA1 genes, all of which have key roles in PCa growth and progression, and are classical AR target genes [51]. Also highly regulated by androgens, fibroblast growth factor 8 (FGF8) [52], cyclin-dependent kinase 1 (CDK1), cyclin-dependent kinase 2 (CDK2) [53], and the gene regulated in breast cancer 1 (GREB1) [54] gene products have classical stimulating roles in prostate growth and proliferation. Conversely, the PMEPA1 gene, although a direct transcriptional target of the AR, has been described as a negative regulator of cell growth in the prostate epithelium, as well as negatively regulating AR protein levels in different cell-culture models [55,56]. We also observed that the AR transcription level was downregulated after PCA3 knockdown. These results accord with previously published data, which demonstrated that the AR gene is transcriptionally regulated by AR through binding to AR regulatory elements (autoregulation). However, differently from the other AR-responsive genes tested here, the ARE elements required for this process have not been found in the AR promoter or in the 5'-flanking region, but rather in AR coding sequences [57]. Since DHT treatment also upregulated AR transcript expression, it is possible that PCA3 could also modulate the transcriptional activity of AR, as has been postulated for other ncRNAs [58,59]. Although it shows both oncogenic and tumor-suppressor roles, PSA also has key roles in promoting tumor progression and metastasis [60]. NDRG1-ERG fusions, encoding a chimeric protein, are also androgen-regulated and correspond to one of the recurrent erythroblast transformation-specific rearrangements found in PCa samples. Presumably, NDRG1 participates in angiogenesis, metastasis, and mechanisms leading to anti-cancer drug resistance [61]. TMPRSS2, another component of these typical androgen-regulated PCa translocations, is highly expressed in PCa cells, contributing to prostate tumorigenesis [62,63]. The observed downregulation of some of these tested AR target genes after PCA3 silencing could be part of the events related to inhibition of PCa cell survival, especially because some of these genes are classical positive modulators of PCa progression. Considering that some of the AR target genes tested were downregulated by PCA3 knockdown, we hypothesize that PCA3, similarly to other ncRNAs, could be a key modulator of the AR signaling pathway, as has been observed for other ncRNA gene products in controlling key pathways [64]. However, highly aggressive metastatic PCa cell lines that are termed AR-insensitive, such as PC3 and DU145, only express PCA3 at very low levels, indicating that PCA3 may play a role (in combination with other factors) by promoting the transition from an AR-dependent to a hormone-refractory disease. Data concerning the expression of AR in PC3 and DU145 cells are contradictory. Although they are classically termed androgen non-responsive and AR-negative cells, the expression of AR transcript and protein has been clearly demonstrated in PC3 and DU145 cells, as has been AR nuclear translocation, but not transcriptional activity [36]. Because PCA3 silencing in PC3 cells also inhibited PC3 viability, it is possible that PCA3 may be involved in AR signaling at alternative steps that are able to control the expression of AR-responsive genes in an androgen-independent manner, as described for other gene targets [65]. Notably, PC3 cells, although they expressed lower PCA3 levels than LNCaP cells, showed greater inhibition of cell survival after PCA3 silencing. Possibly, due to lower PCA3 levels in PC3 cells, silencing of this transcript was more effective, with a stronger negative effect on cell survival.

Based on these data, we suggest that PCA3 probably behaves as a modulator of the expression of the AR target gene, although the underlying mechanism by which PCA3 regulates the expression of these AR target genes remains elusive. One hypothesis is that PCA3 long ncRNA or its putative processed RNA products could directly control the transcription of AR-regulated genes, as has been reported for other ncRNAs [59,66]. It is also possible that PCA3 transcripts could modulate the transcriptional activity of AR-regulated genes by controlling the interaction and/or expression of multiple AR co-regulatory proteins, which facilitate the formation of an active transcription complex to activate transcription of AR target genes, similarly to what has been described for the Steroid Receptor Coactivator (SRA), a non-coding RNA that confers functional specificity during transcriptional activation [67]. Inactive or under-represented co-activators could not evoke AR binding to their cognate binding sites on AR-responsive genes, promoting the downregulation of these genes [68]. This possibility accords with our data regarding the downregulation of the tested AR target genes after PCA3 knockdown, indicating that a key positive AR signal was disrupted. Furthermore, the downregulation of these AR target genes occurred even in LNCaP cells that were not DHT-activated, indicating that the role of PCA3 in modulating the transcription of these genes is independent of AR ligand activation. Our current studies highlight the need for further investigation to elucidate the exact mechanisms by which PCA3 controls PCa cell survival and how it modulates AR signaling and cell growth.

The subcellular location of ncRNAs has also provided significant insights into their functions. We found in the present study that PCA3 is mainly expressed in the nuclear and microsomal subcellular fractions in LNCaP prostate epithelial cells, but not in prostate-tumor stromal cells. Contradictory findings regarding PCA3 expression have indicated that this transcript was restricted to the nuclear [12] or cytoplasmic [13] cell compartments. In order to clarify this, we used a cell-fractionated PCA3 transcript expression analysis approach, in which most PCA3 transcripts were located in nuclear fractions, and to a lesser extent in microsomal compartments, which classically contain ribosomal and vesicle particles. Other long non-coding RNAs have also been found in nuclear fractions, especially accumulated in specific nuclear bodies [69]. Other reports have also identified ncRNAs at nuclear speckles, where these molecules could regulate alternative splicing by modulating the activity of splicing regulatory proteins [70]. Other roles described for ncRNAs located in nuclear compartments, including their roles in modulating the activity of transcription factors and in chromatin remodeling, have been reviewed elsewhere [71]. Co-purification with ribosomes has also been described for other long ncRNAs, such as HULC, implying that they play a role in translational control [72] and in nonsense-mediated mRNA decay (NMD) [73]. The presence of PCA3 transcripts in both nuclear and microsomal compartments may indicate that PCA3 could perform its main roles in both cell niches. These data may help to direct our future approaches in attempting to identify the exact roles of PCA3 in modulating the transcription of AR target genes. Because PCA3 is not expressed in stromal cells, it seems that its role in controlling PCa cell survival may be restricted to prostate epithelial cells and possibly their AR signaling pathway.

## Conclusions

Here we demonstrate for the first time that PCA3 is involved in the control of PCa cell survival, at least in part by modulating the transcriptional activity of AR target genes. To our knowledge, this is the first characterization of the functional role of PCA3 in PCa cells, and will not only improve the understanding of key roles of this transcript in prostate carcinogenesis, but also suggests an alternative strategy to use PCA3 as a putative specific target for PCa treatment approaches. Because PCA3 seems to be a regulator of the expression of AR target genes and PCa cell survival, treatment options aiming to downregulate PCA3, in combination with other androgen-depletion-based strategies, could potentially circumvent androgen-ablation resistance mechanisms.

## Competing interests

The authors declare that they have no competing interests.

## Authors information

Luiz Ricardo Goulart and Etel Rodrigues Pereira Gimba are the co-senior authors.

## Authors' contributions

LFB carried out all the RNA interference assays, all qRTpCR assays, the DHT and AR signaling experiments, participated in the design and analysis of the study, performed the statistical analyses, and drafted the manuscript. KDM carried out the immunoblot assays. AP, MSC, FL and LEN participated in the cell cycle assays and corresponding data analysis. CS and MSC participated in performing and designing the pyknotic nuclei assays. AFN participated in designing siPCA3 sequences and PCA3 oligonucleotides. LRGF conceived the study, participated in its design, and revised the final manuscript. ERPG conceived the study, participated in its design and coordination, and drafted the manuscript. All authors read and approved the final manuscript.

## Pre-publication history

The pre-publication history for this paper can be accessed here:

http://www.biomedcentral.com/1471-2407/12/507/prepub
